# (*E*)-3-(Anthracen-9-yl)-1-(furan-2-yl)prop-2-en-1-one^1^

**DOI:** 10.1107/S1600536810005982

**Published:** 2010-03-13

**Authors:** Jirapa Horkaew, Thitipone Suwunwong, Suchada Chantrapromma, Chatchanok Karalai, Hoong-Kun Fun

**Affiliations:** a Crystal Materials Research Unit, Department of Chemistry, Faculty of Science, Prince of Songkla University, Hat-Yai, Songkhla 90112, Thailand; b X-ray Crystallography Unit, School of Physics, Universiti Sains Malaysia, 11800 USM, Penang, Malaysia

## Abstract

In the mol­ecule of the title heteroaryl chalcone derivative, C_21_H_14_O_2_, the almost planar prop-2-en-1-one unit [r.m.s. deviation = 0.0087 (1) Å] forms dihedral angles of 5.81 (7) and 49.85 (6)°, respectively, with the furan ring and anthracene ring system. In the crystal structure, the mol­ecules are linked into a two-dimensional network parallel to (100) by C—H⋯O hydrogen bonds and π⋯π inter­actions involving the furan rings [centroid–centroid distance = 3.7205 (6) Å].

## Related literature

For background and applications of chalcones, see: Gaber *et al.* (2008 ▶); Niu *et al.* (2006 ▶); Xu *et al.* (2005 ▶). For related structures, see: Chantrapromma *et al.* (2009 ▶, 2010 ▶); Fun *et al.* (2009 ▶); Suwunwong *et al.* (2009 ▶). For bond-length data, see: Allen *et al.* (1987 ▶). For the stability of the temperature controller used in the data collection, see: Cosier & Glazer (1986 ▶).
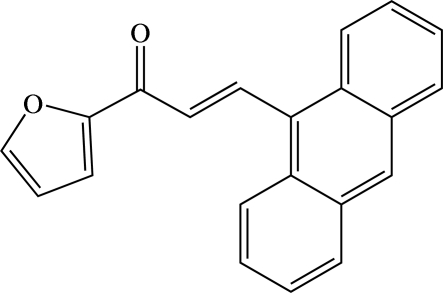


## Experimental

### 

#### Crystal data

C_21_H_14_O_2_
*M* *_r_* = 298.32
Monoclinic, 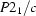

*a* = 21.5743 (4) Å
*b* = 5.4571 (1) Å
*c* = 12.8394 (2) Å
β = 104.099 (1)°
*V* = 1466.09 (4) Å^3^
*Z* = 4
Mo *K*α radiation
μ = 0.09 mm^−1^
*T* = 100 K
0.55 × 0.25 × 0.07 mm

#### Data collection

Bruker APEXII CCD area-detector diffractometer
Absorption correction: multi-scan (*SADABS*; Bruker, 2005 ▶) *T*_min_ = 0.955, *T*_max_ = 0.994
19468 measured reflections
4251 independent reflections
3549 reflections with *I* > 2σ(*I*)
*R*_int_ = 0.029

#### Refinement

*R*[*F* ^2^ > 2σ(*F* ^2^)] = 0.041
*wR*(*F* ^2^) = 0.118
*S* = 1.03
4251 reflections
264 parameters
All H-atom parameters refined
Δρ_max_ = 0.37 e Å^−3^
Δρ_min_ = −0.22 e Å^−3^

### 

Data collection: *APEX2* (Bruker, 2005 ▶); cell refinement: *SAINT* (Bruker, 2005 ▶); data reduction: *SAINT*; program(s) used to solve structure: *SHELXTL* (Sheldrick, 2008 ▶); program(s) used to refine structure: *SHELXTL*; molecular graphics: *SHELXTL*; software used to prepare material for publication: *SHELXTL* and *PLATON* (Spek, 2009 ▶).

## Supplementary Material

Crystal structure: contains datablocks global, I. DOI: 10.1107/S1600536810005982/ci5032sup1.cif

Structure factors: contains datablocks I. DOI: 10.1107/S1600536810005982/ci5032Isup2.hkl

Additional supplementary materials:  crystallographic information; 3D view; checkCIF report

## Figures and Tables

**Table 1:** Hydrogen-bond geometry (Å, °)

*D*—H⋯*A*	*D*—H	H⋯*A*	*D*⋯*A*	*D*—H⋯*A*
C3—H3⋯O1^i^	0.98 (2)	2.34 (2)	3.2871 (14)	165 (1)
C6—H6⋯O1^i^	0.94 (2)	2.40 (2)	3.3366 (13)	173 (1)
C19—H19⋯O1^ii^	0.98 (1)	2.47 (1)	3.3419 (13)	148 (1)

Symmetry codes: (i) 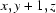; (ii) 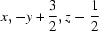.

**Fig. 1.**
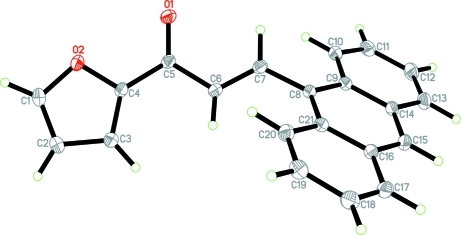
The molecular structure of the title compound, showing 50% probability displacement ellipsoids and the atom-numbering scheme.

**Fig. 2.**
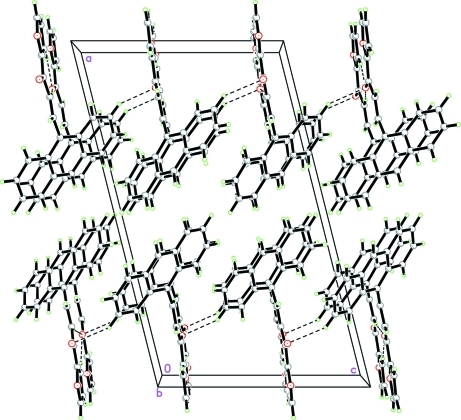
The crystal packing of the title compound viewed along the b axis. C—H···O hydrogen bonds are shown as dashed lines.

## supplementary crystallographic information

### Comment

Chalcones have been studied for their wide range of applications including laser activity (Gaber *et al.*, 2008) and fluorescence properties (Niu *et al.*, 2006; Xu *et al.*, 2005). We have previously reported crystal structures of several chalcone derivatives containing the anthracene moiety which exist in *E* configuration (Suwunwong *et al.*, 2009) or *Z* configuration (Chantrapromma *et al.*, 2009, 2010; Fun *et al.*, 2009). The title compound was synthesized on account of its fluorescence properties. The crystal structure determination was undertaken to elucidate its conformation and to study the structure and fluorescence activity relationship.

The molecule of the title chalcone derivative (Fig. 1) exists in an *E* configuration with respect to the C6═C7 ethenyl bond, with a C5—C6—C7—C8 torsion angle of 178.00 (9)°. The anthracene ring system (C8–C21) is essentially planar (r.m.s. deviation = 0.0258 (1) Å). The prop-2-en-1-one unit (C5–C7/O1) is also planar (r.m.s. deviation = 0.0087 (1) Å; O1—C5—C6—C7 = 2.92 (15)°) and it forms dihedral angles of 5.81 (7) and 49.85 (6)°, respectively, with the furan ring and anthracene ring system. The interplanar angle between the furan ring and anthracene ring system is 48.53 (5)°. The bond distances show normal values (Allen *et al.*, 1987) and are comparable with those in closely related structures (Chantrapromma, Horkaew *et al.*, 2009; Chantrapromma, Suwunwong *et al.*, 2010; Fun *et al.*, 2009; Suwunwong *et al.*, 2009).

In the crystal structure, the molecules are linked into a two-dimensional network parallel to the (100) by C—H···O hydrogen bonds (Fig. 2 and Table 1) and π···π interactions between the furan rings at (x, y, z) and (-x, 1-y, -z) [centroid···centroid distance = 3.7205 (6) Å].

### Experimental

The title compound was synthesized by the condensation of anthracene-9-carbaldehyde (0.41 g, 2 mmol) with 2-furylmethylketone (0.22 g, 2 mmol) in ethanol (30 ml) in the presence of 30 % aqueous NaOH (5 ml) at room temperature. The reaction mixture was stirred at 278 K for 3 h and then a yellow solid appeared was collected by filtration, washed with acetone and dried in air. Yellow plate-shaped single crystals of the title compound suitable for *X*-ray structure determination were recrystalized from acetone-ethanol (1:1 v/v) by slow evaporation of the solvent at room temperature after several days (m.p. 423-424 K).

### Refinement

All H atoms were located in a difference map and refined isotropically [C–H = 0.941 (15)–1.009 (14) Å].

### Crystal data

**Table:**

C_21_H_14_O_2_	*F*(000) = 624
*M**_r_* = 298.32	*D*_x_ = 1.352 Mg m^−^^3^
Monoclinic, *P*2_1_/*c*	Melting point = 423–424 K
Hall symbol: -P 2ybc	Mo *K*α radiation, λ = 0.71073 Å
*a* = 21.5743 (4) Å	Cell parameters from 4251 reflections
*b* = 5.4571 (1) Å	θ = 2.9–30.0°
*c* = 12.8394 (2) Å	µ = 0.09 mm^−^^1^
β = 104.099 (1)°	*T* = 100 K
*V* = 1466.09 (4) Å^3^	Plate, yellow
*Z* = 4	0.55 × 0.25 × 0.07 mm

### Data collection

**Table:**

Bruker APEXII CCD area-detector diffractometer	4251 independent reflections
Radiation source: sealed tube	3549 reflections with *I* > 2σ(*I*)
graphite	*R*_int_ = 0.029
φ and ω scans	θ_max_ = 30.0°, θ_min_ = 2.9°
Absorption correction: multi-scan (*SADABS*; Bruker, 2005)	*h* = −26→30
*T*_min_ = 0.955, *T*_max_ = 0.994	*k* = −7→7
19468 measured reflections	*l* = −17→18

### Refinement

**Table:**

Refinement on *F*^2^	Primary atom site location: structure-invariant direct methods
Least-squares matrix: full	Secondary atom site location: difference Fourier map
*R*[*F*^2^ > 2σ(*F*^2^)] = 0.041	Hydrogen site location: inferred from neighbouring sites
*wR*(*F*^2^) = 0.118	All H-atom parameters refined
*S* = 1.03	*w* = 1/[σ^2^(*F*_o_^2^) + (0.0624*P*)^2^ + 0.4737*P*] where *P* = (*F*_o_^2^ + 2*F*_c_^2^)/3
4251 reflections	(Δ/σ)_max_ = 0.001
264 parameters	Δρ_max_ = 0.37 e Å^−^^3^
0 restraints	Δρ_min_ = −0.22 e Å^−^^3^

### Special details

**Table:**

Experimental. The crystal was placed in the cold stream of an Oxford Cryosystems Cobra open-flow nitrogen cryostat (Cosier & Glazer, 1986) operating at 120.0 (1) K.
Geometry. All esds (except the esd in the dihedral angle between two l.s. planes) are estimated using the full covariance matrix. The cell esds are taken into account individually in the estimation of esds in distances, angles and torsion angles; correlations between esds in cell parameters are only used when they are defined by crystal symmetry. An approximate (isotropic) treatment of cell esds is used for estimating esds involving l.s. planes.
Refinement. Refinement of *F*^2^ against ALL reflections. The weighted *R*-factor wR and goodness of fit *S* are based on *F*^2^, conventional *R*-factors *R* are based on *F*, with *F* set to zero for negative *F*^2^. The threshold expression of *F*^2^ > σ(*F*^2^) is used only for calculating *R*-factors(gt) etc. and is not relevant to the choice of reflections for refinement. *R*-factors based on *F*^2^ are statistically about twice as large as those based on *F*, and *R*- factors based on ALL data will be even larger.

### Fractional atomic coordinates and isotropic or equivalent isotropic displacement parameters (Å^2^)

**Table:**

	*x*	*y*	*z*	*U*_iso_*/*U*_eq_	
O1	0.13071 (4)	0.32065 (14)	0.15881 (6)	0.02003 (17)	
O2	0.00592 (3)	0.46137 (14)	0.12589 (6)	0.01846 (17)	
C1	−0.04942 (5)	0.5903 (2)	0.11274 (9)	0.0201 (2)	
H1	−0.0880 (7)	0.489 (3)	0.1018 (12)	0.030 (4)*	
C2	−0.03863 (5)	0.8346 (2)	0.11454 (9)	0.0208 (2)	
H2	−0.0701 (8)	0.964 (3)	0.1084 (13)	0.034 (4)*	
C3	0.02854 (5)	0.8641 (2)	0.12983 (8)	0.0177 (2)	
H3	0.0517 (7)	1.019 (3)	0.1342 (11)	0.027 (4)*	
C4	0.05367 (5)	0.63332 (19)	0.13651 (8)	0.0152 (2)	
C5	0.11877 (5)	0.54141 (19)	0.15040 (8)	0.0150 (2)	
C6	0.16862 (5)	0.72918 (19)	0.15159 (8)	0.0163 (2)	
C7	0.22914 (5)	0.66214 (19)	0.15739 (8)	0.0159 (2)	
C8	0.28183 (5)	0.83333 (19)	0.15569 (8)	0.01475 (19)	
C9	0.34004 (5)	0.80973 (19)	0.23544 (8)	0.0153 (2)	
C10	0.34956 (5)	0.6249 (2)	0.31680 (8)	0.0183 (2)	
C11	0.40571 (5)	0.6100 (2)	0.39433 (9)	0.0214 (2)	
C12	0.45582 (5)	0.7804 (2)	0.39679 (9)	0.0231 (2)	
C13	0.44862 (5)	0.9614 (2)	0.32146 (9)	0.0214 (2)	
C14	0.39083 (5)	0.9814 (2)	0.23836 (8)	0.0166 (2)	
C15	0.38256 (5)	1.1693 (2)	0.16251 (8)	0.0181 (2)	
C16	0.32655 (5)	1.19033 (19)	0.08122 (8)	0.0164 (2)	
C17	0.31916 (5)	1.3816 (2)	0.00291 (9)	0.0201 (2)	
C18	0.26502 (6)	1.3994 (2)	−0.07801 (9)	0.0218 (2)	
C19	0.21559 (5)	1.2226 (2)	−0.08673 (8)	0.0209 (2)	
C20	0.22049 (5)	1.0396 (2)	−0.01267 (8)	0.0183 (2)	
C21	0.27525 (5)	1.01872 (19)	0.07639 (8)	0.0154 (2)	
H6	0.1563 (7)	0.895 (3)	0.1471 (11)	0.021 (3)*	
H7	0.2399 (6)	0.482 (3)	0.1653 (11)	0.019 (3)*	
H10	0.3158 (7)	0.507 (3)	0.3176 (11)	0.024 (4)*	
H11	0.4105 (8)	0.479 (3)	0.4498 (13)	0.035 (4)*	
H12	0.4960 (7)	0.766 (3)	0.4544 (13)	0.033 (4)*	
H13	0.4831 (8)	1.086 (3)	0.3216 (13)	0.032 (4)*	
H15	0.4171 (7)	1.291 (3)	0.1670 (11)	0.024 (4)*	
H17	0.3534 (7)	1.501 (3)	0.0097 (12)	0.027 (4)*	
H18	0.2611 (8)	1.533 (3)	−0.1306 (13)	0.036 (4)*	
H19	0.1780 (7)	1.232 (3)	−0.1469 (11)	0.025 (4)*	
H20	0.1864 (7)	0.916 (3)	−0.0210 (11)	0.025 (4)*	

### Atomic displacement parameters (Å^2^)

**Table:**

	*U*^11^	*U*^22^	*U*^33^	*U*^12^	*U*^13^	*U*^23^
O1	0.0184 (4)	0.0134 (4)	0.0265 (4)	−0.0007 (3)	0.0020 (3)	0.0008 (3)
O2	0.0144 (3)	0.0166 (4)	0.0236 (4)	−0.0032 (3)	0.0031 (3)	0.0005 (3)
C1	0.0134 (5)	0.0248 (5)	0.0216 (5)	−0.0011 (4)	0.0031 (4)	0.0001 (4)
C2	0.0154 (5)	0.0222 (5)	0.0244 (5)	0.0021 (4)	0.0042 (4)	0.0002 (4)
C3	0.0165 (5)	0.0165 (5)	0.0196 (5)	−0.0007 (4)	0.0034 (4)	−0.0002 (4)
C4	0.0142 (4)	0.0148 (5)	0.0161 (4)	−0.0026 (4)	0.0027 (3)	0.0000 (3)
C5	0.0147 (4)	0.0143 (5)	0.0149 (4)	−0.0017 (3)	0.0016 (3)	−0.0006 (3)
C6	0.0161 (5)	0.0125 (4)	0.0199 (4)	−0.0013 (4)	0.0038 (4)	−0.0004 (4)
C7	0.0163 (5)	0.0139 (5)	0.0170 (4)	−0.0011 (4)	0.0030 (3)	−0.0004 (3)
C8	0.0136 (4)	0.0137 (4)	0.0175 (4)	−0.0003 (3)	0.0047 (3)	−0.0019 (3)
C9	0.0140 (4)	0.0155 (5)	0.0173 (4)	0.0008 (4)	0.0051 (3)	−0.0012 (4)
C10	0.0162 (5)	0.0180 (5)	0.0212 (5)	0.0012 (4)	0.0054 (4)	0.0007 (4)
C11	0.0190 (5)	0.0232 (5)	0.0219 (5)	0.0047 (4)	0.0049 (4)	0.0032 (4)
C12	0.0156 (5)	0.0286 (6)	0.0232 (5)	0.0024 (4)	0.0011 (4)	0.0001 (4)
C13	0.0139 (5)	0.0248 (6)	0.0245 (5)	−0.0009 (4)	0.0027 (4)	−0.0015 (4)
C14	0.0135 (4)	0.0177 (5)	0.0192 (4)	−0.0002 (4)	0.0050 (4)	−0.0026 (4)
C15	0.0157 (5)	0.0184 (5)	0.0213 (5)	−0.0025 (4)	0.0067 (4)	−0.0012 (4)
C16	0.0167 (5)	0.0153 (5)	0.0190 (4)	−0.0003 (4)	0.0075 (4)	−0.0014 (4)
C17	0.0217 (5)	0.0176 (5)	0.0234 (5)	−0.0010 (4)	0.0105 (4)	0.0013 (4)
C18	0.0259 (6)	0.0213 (5)	0.0207 (5)	0.0027 (4)	0.0106 (4)	0.0039 (4)
C19	0.0204 (5)	0.0243 (6)	0.0175 (5)	0.0024 (4)	0.0038 (4)	0.0011 (4)
C20	0.0170 (5)	0.0193 (5)	0.0184 (4)	−0.0003 (4)	0.0037 (4)	−0.0003 (4)
C21	0.0150 (4)	0.0149 (5)	0.0168 (4)	0.0006 (4)	0.0049 (3)	−0.0011 (4)

### Geometric parameters (Å, °)

**Table:**

O1—C5	1.2312 (13)	C10—H10	0.973 (15)
O2—C1	1.3606 (13)	C11—C12	1.4209 (17)
O2—C4	1.3754 (12)	C11—H11	0.997 (17)
C1—C2	1.3526 (16)	C12—C13	1.3642 (17)
C1—H1	0.981 (16)	C12—H12	0.996 (16)
C2—C3	1.4235 (15)	C13—C14	1.4342 (14)
C2—H2	0.968 (17)	C13—H13	1.007 (16)
C3—C4	1.3654 (14)	C14—C15	1.3950 (15)
C3—H3	0.974 (15)	C15—C16	1.3959 (14)
C4—C5	1.4609 (14)	C15—H15	0.988 (15)
C5—C6	1.4827 (14)	C16—C17	1.4310 (15)
C6—C7	1.3406 (14)	C16—C21	1.4396 (14)
C6—H6	0.941 (15)	C17—C18	1.3645 (16)
C7—C8	1.4757 (14)	C17—H17	0.972 (15)
C7—H7	1.009 (14)	C18—C19	1.4225 (17)
C8—C21	1.4176 (14)	C18—H18	0.984 (17)
C8—C9	1.4196 (14)	C19—C20	1.3653 (15)
C9—C10	1.4303 (14)	C19—H19	0.975 (14)
C9—C14	1.4353 (14)	C20—C21	1.4346 (14)
C10—C11	1.3701 (15)	C20—H20	0.984 (15)
			
C1—O2—C4	105.84 (8)	C10—C11—H11	118.7 (9)
C2—C1—O2	111.43 (10)	C12—C11—H11	120.6 (9)
C2—C1—H1	134.0 (9)	C13—C12—C11	120.23 (10)
O2—C1—H1	114.6 (9)	C13—C12—H12	120.8 (9)
C1—C2—C3	106.20 (10)	C11—C12—H12	119.0 (9)
C1—C2—H2	127.0 (10)	C12—C13—C14	120.72 (10)
C3—C2—H2	126.8 (10)	C12—C13—H13	122.1 (9)
C4—C3—C2	106.24 (9)	C14—C13—H13	117.2 (9)
C4—C3—H3	127.2 (9)	C15—C14—C13	121.04 (10)
C2—C3—H3	126.6 (9)	C15—C14—C9	119.63 (9)
C3—C4—O2	110.29 (9)	C13—C14—C9	119.30 (10)
C3—C4—C5	132.80 (9)	C14—C15—C16	121.51 (10)
O2—C4—C5	116.90 (9)	C14—C15—H15	119.0 (8)
O1—C5—C4	121.40 (9)	C16—C15—H15	119.5 (8)
O1—C5—C6	122.66 (9)	C15—C16—C17	120.89 (10)
C4—C5—C6	115.93 (9)	C15—C16—C21	119.67 (9)
C7—C6—C5	120.41 (10)	C17—C16—C21	119.43 (9)
C7—C6—H6	121.6 (9)	C18—C17—C16	121.01 (10)
C5—C6—H6	118.0 (9)	C18—C17—H17	120.7 (9)
C6—C7—C8	124.74 (10)	C16—C17—H17	118.3 (9)
C6—C7—H7	117.9 (8)	C17—C18—C19	119.86 (10)
C8—C7—H7	117.4 (8)	C17—C18—H18	119.4 (9)
C21—C8—C9	119.94 (9)	C19—C18—H18	120.8 (9)
C21—C8—C7	121.30 (9)	C20—C19—C18	120.81 (10)
C9—C8—C7	118.76 (9)	C20—C19—H19	120.0 (9)
C8—C9—C10	122.36 (9)	C18—C19—H19	119.2 (9)
C8—C9—C14	119.68 (9)	C19—C20—C21	121.48 (10)
C10—C9—C14	117.92 (9)	C19—C20—H20	119.7 (8)
C11—C10—C9	121.13 (10)	C21—C20—H20	118.8 (8)
C11—C10—H10	118.9 (8)	C8—C21—C20	123.23 (9)
C9—C10—H10	119.9 (8)	C8—C21—C16	119.46 (9)
C10—C11—C12	120.68 (10)	C20—C21—C16	117.25 (9)
			
C4—O2—C1—C2	0.05 (12)	C12—C13—C14—C15	178.54 (10)
O2—C1—C2—C3	0.07 (13)	C12—C13—C14—C9	0.46 (16)
C1—C2—C3—C4	−0.16 (12)	C8—C9—C14—C15	−0.25 (15)
C2—C3—C4—O2	0.20 (11)	C10—C9—C14—C15	−177.91 (9)
C2—C3—C4—C5	179.04 (10)	C8—C9—C14—C13	177.85 (9)
C1—O2—C4—C3	−0.16 (11)	C10—C9—C14—C13	0.20 (15)
C1—O2—C4—C5	−179.20 (8)	C13—C14—C15—C16	179.91 (10)
C3—C4—C5—O1	176.89 (11)	C9—C14—C15—C16	−2.01 (16)
O2—C4—C5—O1	−4.33 (14)	C14—C15—C16—C17	−178.97 (10)
C3—C4—C5—C6	−4.22 (16)	C14—C15—C16—C21	1.51 (15)
O2—C4—C5—C6	174.56 (8)	C15—C16—C17—C18	178.56 (10)
O1—C5—C6—C7	2.92 (15)	C21—C16—C17—C18	−1.92 (16)
C4—C5—C6—C7	−175.95 (9)	C16—C17—C18—C19	−1.51 (17)
C5—C6—C7—C8	178.00 (9)	C17—C18—C19—C20	2.45 (17)
C6—C7—C8—C21	−49.44 (15)	C18—C19—C20—C21	0.14 (17)
C6—C7—C8—C9	130.63 (11)	C9—C8—C21—C20	173.77 (9)
C21—C8—C9—C10	−179.46 (9)	C7—C8—C21—C20	−6.16 (15)
C7—C8—C9—C10	0.48 (15)	C9—C8—C21—C16	−3.49 (15)
C21—C8—C9—C14	2.99 (15)	C7—C8—C21—C16	176.57 (9)
C7—C8—C9—C14	−177.07 (9)	C19—C20—C21—C8	179.21 (10)
C8—C9—C10—C11	−178.45 (10)	C19—C20—C21—C16	−3.47 (15)
C14—C9—C10—C11	−0.86 (15)	C15—C16—C21—C8	1.27 (15)
C9—C10—C11—C12	0.87 (17)	C17—C16—C21—C8	−178.26 (9)
C10—C11—C12—C13	−0.18 (18)	C15—C16—C21—C20	−176.16 (9)
C11—C12—C13—C14	−0.48 (18)	C17—C16—C21—C20	4.31 (14)

### Hydrogen-bond geometry (Å, °)

**Table:**

*D*—H···*A*	*D*—H	H···*A*	*D*···*A*	*D*—H···*A*
C3—H3···O1^i^	0.98 (2)	2.34 (2)	3.2871 (14)	165 (1)
C6—H6···O1^i^	0.94 (2)	2.40 (2)	3.3366 (13)	173 (1)
C19—H19···O1^ii^	0.98 (1)	2.47 (1)	3.3419 (13)	148 (1)

Symmetry codes: (i) *x*, *y*+1, *z*; (ii) *x*, −*y*+3/2, *z*−1/2.
